# N-terminal Huntingtin (Htt) phosphorylation is a molecular switch regulating Htt aggregation, helical conformation, internalization, and nuclear targeting

**DOI:** 10.1074/jbc.RA118.004621

**Published:** 2018-09-05

**Authors:** Sean M. DeGuire, Francesco S. Ruggeri, Mohamed-Bilal Fares, Anass Chiki, Urszula Cendrowska, Giovanni Dietler, Hilal A. Lashuel

**Affiliations:** From the ‡ Laboratory of Molecular and Chemical Biology of Neurodegeneration, Brain Mind Institute, École Polytechnique Fédérale de Lausanne (EPFL), CH-1015 Lausanne, Switzerland and; the § Laboratory of the Physics of Living Matter, Institute of Physics of Biological Systems, École Polytechnique Fédérale de Lausanne (EPFL), CH-1015 Lausanne, Switzerland

**Keywords:** Huntington's disease, aggregation, phosphorylation, post-translational modification (PTM), neurodegenerative disease, Amyloid, Htt protein, Httex1, phosphomimetic

## Abstract

Huntington's disease is a fatal neurodegenerative disorder resulting from a CAG repeat expansion in the first exon of the gene encoding the Huntingtin protein (Htt). Phosphorylation of this protein region (Httex1) has been shown to play important roles in regulating the structure, toxicity, and cellular properties of N-terminal fragments and full-length Htt. However, increasing evidence suggests that phosphomimetic substitutions in Htt result in inconsistent findings and do not reproduce all aspects of true phosphorylation. Here, we investigated the effects of *bona fide* phosphorylation at Ser-13 or Ser-16 on the structure, aggregation, membrane binding, and subcellular properties of the Httex1-Q18A variant and compared these effects with those of phosphomimetic substitutions. We show that phosphorylation at either Ser-13 and/or Ser-16 or phosphomimetic substitutions at both these residues inhibit the aggregation of mutant Httex1, but that only phosphorylation strongly disrupts the amphipathic α-helix of the N terminus and prompts the internalization and nuclear targeting of preformed Httex1 aggregates. In synthetic peptides, phosphorylation at Ser-13, Ser-16, or both residues strongly disrupted the amphipathic α-helix of the N-terminal 17 residues (Nt17) of Httex1 and Nt17 membrane binding. Experiments with peptides bearing different combinations of phosphorylation sites within Nt17 revealed a phosphorylation-dependent switch that regulates the Httex1 structure, involving cross-talk between phosphorylation at Thr-3 and Ser-13 or Ser-16. Our results provide crucial insights into the role of phosphorylation in regulating Httex1 structure and function, and underscore the critical importance of identifying the enzymes responsible for regulating Htt phosphorylation, and their potential as therapeutic targets for managing Huntington's disease.

## Introduction

Huntington's disease (HD)^3^ is a fatal neurodegenerative disorder that results from a CAG repeat expansion (>36) in the first exon of the *IT-15* gene encoding the Huntingtin protein (*HTT*) (1). Although the length of the CAG-encoded polyQ tract seems to directly correlate with disease severity and Htt protein aggregation propensity (2–4), other sequence features, including post-translational modifications (PTMs), have been shown to influence the aggregation, cellular properties, and toxicity of *HTT* and therefore may contribute to HD pathogenesis (5–11). The identification of PTMs that promote or protect against Htt-induced toxicity could lead to the development of new drugs for the treatment of HD by modulating these modifications and their downstream effects.

N-terminal fragments of Htt have been found in nuclear inclusion bodies in HD patients (12), and overexpression of exon 1 of Htt (Httex1) with expanded polyQ repeats alone is sufficient to a variable extent recapitulate many characteristics of HD in mice (13), suggesting that N-terminal Htt fragments may play a critical role in the pathogenesis of HD (14, 15). These observations, combined with the fact that these fragments represent more tractable systems for structure–function studies, have made N-terminal Htt fragments the focus of many studies aimed at elucidating the structural basis of Htt aggregation and toxicity. Httex1 is composed of three domains: the Nt17 domain comprising the first 17 amino acids, the polyQ domain, and the proline-rich domain, all of which play important roles in the misfolding, oligomerization, and/or fibrillization of Httex1 and full-length Htt (4, 16–22). *In vitro* biophysical studies suggest that Httex1 aggregation, although polyQ-dependent, occurs through a two-step mechanism that involves an association of the Nt17 domain to form homooligomeric species characterized by increased α-helical content, which then undergoes conformational changes to form β-sheet–rich fibrils that grow through monomer addition (23). Other studies suggest that the Nt17 interaction with the polyQ domain influences the structure of Httex1 in ways that favor the population of aggregation-prone conformational intermediates (17, 67). Consistent with these studies, N-terminal Htt fragments lacking Nt17 show reduced aggregation rates compared with those containing Nt17 with similar polyQ repeat lengths (19). In addition to its role in Htt aggregation, this domain has been implicated in the regulation of full-length Htt protein–protein interactions (21, 27–30), subcellular localization (10), and toxicity (31).

The Nt17 domain comprises several residues that have been shown to undergo phosphorylation (Thr-3 (5, 24, 63), Ser-13, and Ser-16 (6, 32)) or other post-translational modifications, such as acetylation (5), ubiquitination, and SUMOylation (33) (Lys-6, Lys-9, and Lys-15) in HD brains, transgenic animals, and cell culture models of HD. Increasing evidence suggests that these PTMs influence Htt structure, aggregation (23, 31), subcellular localization (32, 34), toxicity (5), and protein–protein interactions (6) and may represent important molecular switches for regulating these properties in health and disease (35). However, dissecting the specific effects of single or multiple modifications has been challenging, because the main enzymes involved in regulating many of these modifications remain unknown. For example, serine phosphorylation of Httex1 has been reported to be mediated by IκB kinase (6) and casein kinase 2 (32); however, the efficiency and selectivity of these enzymes are too poor to make them reliable tools for efficient manipulation of phosphorylation at these residues *in vivo* or for the generation of homogeneously phosphorylated recombinant Htt proteins *in vitro*. Because of these challenges, most studies investigating the effects of phosphorylation to date have relied on the overexpression of phosphomimetic (S→D/E) or phosphoresistant (S→A) mutant forms of Htt. In cell culture models, the introduction of phosphomimetic mutations at both Ser-13 and Ser-16 has been consistently shown to modify the levels of Httex1 ubiquitination and acetylation, increase Htt nuclear localization (32), reduce its toxicity, and influence its clearance (6). However, overexpression of these mutants in animal models of HD has led to conflicting results. For example, the overexpression in *Drosophila* of (S13D/S16D) 120Q mutant Htt (mHtt) enhanced toxicity compared with unmodified mHtt (36, 37), whereas the introduction of the S13D/S16D mutations in full-length Htt with expanded polyQ repeats (97Q) in a BACHD transgenic mouse model reversed mutant Htt–induced pathology and disease phenotype. In terms of motor deficits, psychiatric-like deficits, and aggregate formation, Ser → Asp mutant BACHD mice were indistinguishable from nontransgenic mice (31). Moreover, recent studies showed that overexpression of single phosphomimetic mutations of Ser-13 and Ser-16 in human cells (using a bimolecular fluorescence complementation assay) or in *Drosophila* was sufficient to inhibit or abolish the aggregation of Httex1 (25), and inhibition of PP1 phosphatase, which modulates Nt17 phosphorylation, was shown to decrease both the aggregation and neurotoxicity (25). However, even in the same study, the effect of the phosphomimetics varied, depending on the expression system used, with phosphomimicking mutations at Ser-13 and Ser-16 leading to decreased or increased aggregation, depending on whether they were expressed in the larval eye imaginal discs of *Drosophila* or in adult dopaminergic neurons. The discrepancy between these studies could be explained by the fact that mutations to mimic or abolish phosphorylation does not allow for assessing the dynamics of phosphorylation and the role of potential cross-talk between the phosphorylation at Thr-3, Ser-13, and Ser-16. Furthermore, increasing evidence shows that the phosphomimicking mutations do not reproduce all aspects of protein phosphorylation (24, 39–41). For example, we recently showed that the T3D mutation did not fully reproduce the inhibitory effect of Thr-3 phosphorylation on the aggregation of Httex1 (24).

To address these limitations, we took advantage of recent advances made by our group that enable the site-specific introduction of post-translational modifications within the Nt17 domain and in the context of a tag-free native WT (22Q) (42) and mutant (42Q) Httex1 using protein semisynthesis (24). We used these strategies to produce, in milligram quantities, single- and double-phosphorylated (pSer-13, pSer-16, and pSer-13/pSer-16) forms of Httex1 with polyQ repeats both above and below the pathogenic threshold of HD. With these proteins in hand, we performed systematic studies to address the following questions. 1) What is the effect of phosphorylation at each or both serine residues on the aggregation and fibril morphology of Httex1? 2) Is the effect of serine phosphorylation dependent on the polyQ repeats' length? 3) Does the introduction of phosphomimetic mutations at Ser-13 and/or Ser-16 reproduce the effects of phosphorylation at these residues on Httex1 secondary structure, aggregation, and membrane binding? 4) How does phosphorylation at Ser-13 and/or Ser-16 affect the internalization and subcellular targeting of Httex1 aggregates in primary neurons?

## Results

### Semisynthesis of Httex1 protein

**Figure 1.**
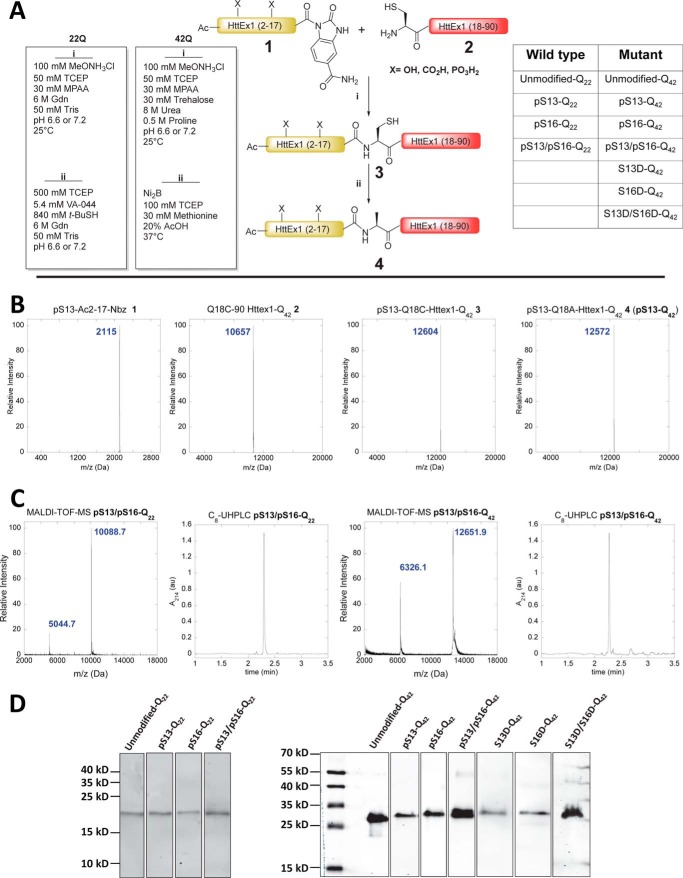
**Semisynthesis of Httex1 phosphorylated at Ser-13 and/or Ser-16.** *A*, *schematic depiction* of the semisynthetic strategy used to produce phosphorylated (pSer) WT (22Q) and mutant (42Q) Q18A Httex1 and the corresponding phosphomimicking mutants. *B*, ESI-MS analyses of starting materials, intermediates, and products of native chemical ligation and desulfurization for pSer-13-Q_42_. *C*, final purity characterization of Httex1 pSer-13/pSer-16-Q_22_ and Httex1 pSer-13/pSer-16-Q_42_ using MALDI-TOF MS and UHPLC. *D*, Western blot analysis characterization of the proteins produced using this semisynthetic strategy and listed in *A*.

**Figure 2.**
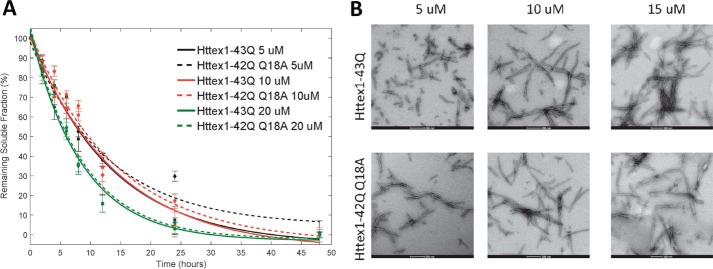
**The substitution of Gln-18 by alanine (Q18A) to facilitate the semisynthesis of phosphorylated Httex1 proteins via NCL does not alter the kinetics of aggregation or morphology of the fibrils formed by mutant Httex1.** *A*, comparison of the aggregation kinetics of Httex1–43Q and Httex1–42Q bearing the Gln → Ala mutation at residue 18 (Q18A) at 5, 10, and 20 μm, as determined by the UHPLC sedimentation assay (*error bars*, S.D. derived from *n* = 3 independent experiments). *B*, TEM analysis of Httex1–43Q and Httex1–42Q (Q18A) at different concentrations (*scale bars*, 200 nm).

Expressed protein ligation requires a nucleophilic cysteine residue at the N terminus of the C-terminal ligation fragment and a thioester at the C terminus of the N-terminal fragment. Httex1 does not contain cysteine residues. In such cases, a transient alanine-to-cysteine mutation is usually introduced to create a ligation site, and the native alanine residue and native protein sequence can be restored by reductive desulfurization of the ligated product. However, no alanine residues exist between the desired phosphorylation sites (Ser-13 and Ser-16) and the polyQ repeat domain. Therefore, we created a ligation site by substituting the first glutamine residue of the polyQ domain with a cysteine (Q18C) to generate WT (22Q) and mutant (42Q) Httex1. After desulfurization, this leads to the introduction of a Gln → Ala mutation at residue 18 (Q18A) in all proteins generated using this semisynthetic strategy. In the absence of kinases that allow for site-specific phosphorylation at Ser-13 and/or Ser-16, this is the only way to introduce site-specific phosphorylation with minimal perturbation of the native sequence (Fig. 1*A*). Therefore, as a control, Httex1 proteins without serine modifications, but bearing this mutation (Q18A-Httex1; unmodified Httex1-Q_22/42_) were prepared using the same semisynthetic strategy outlined above and were always used for direct comparison with the modified proteins in all of our biophysical studies. To ensure that the presence of the Q18A mutation does not affect the aggregation kinetics of mutant Httex1, we compared the aggregation of Httex1–42Q Q18A with the aggregation of Httex1–43Q at different concentrations (5, 10, and 20 μm). Fig. 2*A* shows that the aggregation kinetics as determined by monitoring the loss of Httex1 monomers are not affected by the Q18A mutation. In addition, examination of the fibrils formed by Httex1–43Q and Httex1–42Q Q18A revealed virtually identical fibril morphologies for both proteins (Fig. 2*B*).

To elucidate the role of phosphorylation at these residues, we generated Httex1 phosphorylated at single serine residues (pSer-13 or pSer-16) and both residues (pSer-13/pSer-16). To determine whether Ser → Asp substitutions would reproduce the effect of phosphorylation at these sites, we also prepared proteins with the corresponding phosphomimetic mutations S13D, S16D, and S13D/S16D using the same semisynthetic strategy. Fig. 1*A* shows a schematic depiction of our semisynthetic strategy and a list of the semisynthetic Httex1 proteins generated and used in this study.

To produce the proteins required for this study, a series of seven N-terminal peptides were prepared as *N*-acyl-benzimidazolinones (NBz) that incorporated the desired phosphorylated residue(s) (43). Previous studies have shown that Httex1 undergoes methionine removal and subsequent A2 acetylation *in vivo* (5). Consequently, all Nt17 peptides used in this study were N-terminally acetylated. To prepare WT (22Q) Httex1 proteins, the Q18C 18–90 22Q Httex1 fragment was expressed in *Escherichia coli* and subsequently purified using an intein-based expression strategy that was recently developed in our laboratory for the production of Httex1 (24). N-terminal thiazolidine products produced from the undesired condensation of cellular electrophiles were removed by hydrolysis at low pH and at 37 °C in the presence of excess methoxyamine (44). Native chemical ligation (NCL) reactions of this fragment (**2** in Fig. 1) with each peptide (**1**) were conducted under reducing and denaturing conditions at neutral pH in the presence of 4-mercaptophenylacetic acid (MPAA) and yielded the desired ligation products at room temperature (24, 42). Unexpectedly, under these conditions, the NBz group of peptides containing Ser-16 phosphorylation was found to convert rapidly to side products via hydrolysis and aminolysis before significant product formation was observed. For ligations with pSer-16 and pSer-13/pSer-16 peptides, the omission of methoxyamine treatment prevented aminolysis, and the slow addition of undiluted peptide–NBz (2.0 eq over 4 h) maintained sufficient amounts of active thioester for NCL to proceed rapidly. Each ligated protein was then treated with radical reductive desulfurization conditions (45). The resulting Q18A Httex1 proteins were purified by RP-HPLC, and their purity was established by SDS-PAGE, reverse-phase ultra-HPLC, LC-MS, and MALDI-TOF MS.

Similarly, mutant Httex1 proteins were prepared by native chemical ligation of the same peptides with recombinant Q18C 18–90 Httex1 (**2**) containing an expanded polyQ repeat (42Q). These ligations were more challenging because the unreacted recombinant C-terminal fragment (**2**) and the ligation products (**3**) exhibited an increased tendency to aggregate during the ligation and desulfurization reactions before completion of the desired transformations. To overcome these challenges, we utilized ligation conditions that we have recently developed by combining strongly denaturing conditions with additives that are known to inhibit protein and polyQ peptide aggregation (24). Under these conditions, the ligation reactions proceeded to completion before the aggregation of the proteins could occur. Nickel boride–mediated reductive desulfurization was found to be compatible with an acidic water/organic solvent system that keeps Httex1 protein soluble even at 37 °C, the temperature required for efficient reduction (46). The purity and chemical identity of the mutant proteins after RP-HPLC purification was established by SDS-PAGE, reverse-phase ultra-HPLC, electrospray ionization (ESI)-MS, and MALDI-TOF MS (Fig. 1).

### Single phosphorylation at Ser-13 or Ser-16 slows the aggregation of mutant Httex1

To evaluate whether single phosphorylation or phosphomimetic mutations at Ser-13 or Ser-16 inhibit the aggregation of mutant (42Q) Httex1, we compared the aggregation properties of unmodified Httex1-Q_42_, pSer-13 Httex1-Q_42_, pSer-16 Httex1-Q_42_, S13D Httex1-Q_42_, and S16D Httex1-Q_42_ Httex1 proteins. The lyophilized proteins were first treated with a previously established disaggregation protocol to allow for solvation in aqueous buffer and to disrupt any preformed aggregates (47). The proteins were then dissolved in PBS, the pH was adjusted to 7.3, and any remaining preformed aggregates were removed by filtration (100-kDa cutoff filter, Merck Millipore). The concentration of each filtrate was adjusted to 2.5 μm using a polyQ repeat length–specific calibration curve based on the UHPLC profiles of samples of known concentration as determined by amino acid analysis (48). The aggregation of the proteins was assessed using a sedimentation assay and monitoring the loss of soluble protein. Sample aliquots were periodically collected over the course of several days from solutions incubated at 37 °C. Following the removal of Httex1 aggregates from these aliquots via sedimentation, the remaining fraction of soluble protein was measured by quantitative UHPLC analysis of the supernatant relative to the initial protein concentration.

**Figure 3.**
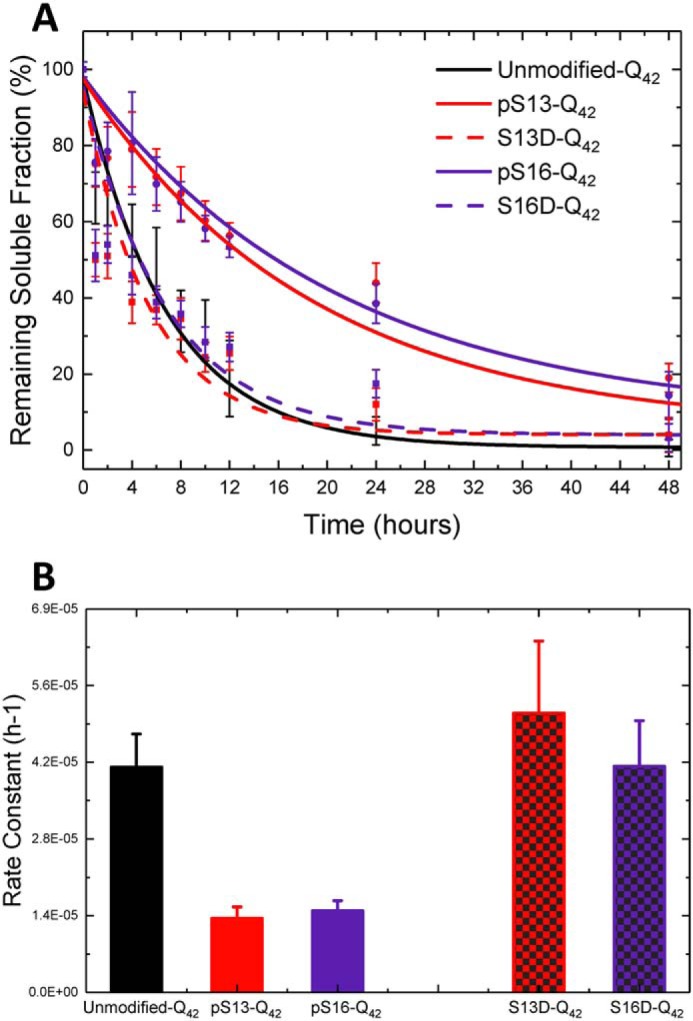
**Phosphorylation at Ser-13 or Ser-16, but not pseudophosphorylation, results in a significant retardation of mutant Httex1 aggregation.** *A*, aggregation analysis of single-phosphorylated and phosphomimetic mutant (42Q) Httex1 Q18A (pSer-13, pSer-16, S13D, and S16D) at 2.5 μm using the UHPLC sedimentation assay (*error bars*, S.D. derived from *n* = 6 for unmodified, *n* = 5 for pSer-13, *n* = 8 for S13D, *n* = 5 for pSer-16, *n* = 8 for S13D, *n* = 4 for pSer-13/pSer-16, and *n* = 8 for S13D/S16D). *B*, calculated aggregation rates.

Complete aggregation and conversion of monomeric unmodified Httex1-Q_42_ into insoluble aggregates occurred within 24 h (Fig. 3*A*). At this time point, all phosphorylated mutant Httex1 proteins showed reduced aggregation compared with the unmodified Httex1, with pSer-13 Httex1-Q_42_ and pSer-16 Httex1-Q_42_ proteins being ∼40% aggregated and only 80% of the proteins converted into insoluble aggregates after 48 h. To obtain an estimate of the rate of aggregation (loss of monomer), the data obtained for each protein aggregation assay were fit to an exponential decay function (48–50). The effect of the single phosphorylation is confirmed when the approximate relative aggregation rates are calculated from the sedimentation data; phosphorylation of either serine (for pSer-13 Httex1-Q_42_ and pSer-16 Httex1-Q_42_) resulted in aggregation rates only 30–35% of the rate of unmodified-Q_42_ (Fig. 3*B*). This inhibitory effect was not reproduced by the Ser → Asp substitutions at these residues. The Httex1 phosphomimetics S13D Httex1-Q_42_ and S16D Httex1-Q_42_ exhibited similar aggregation rates and degrees of aggregation as the unmodified Httex1-Q_42_ (Fig. 3, *A* and *B*).

**Figure 4.**
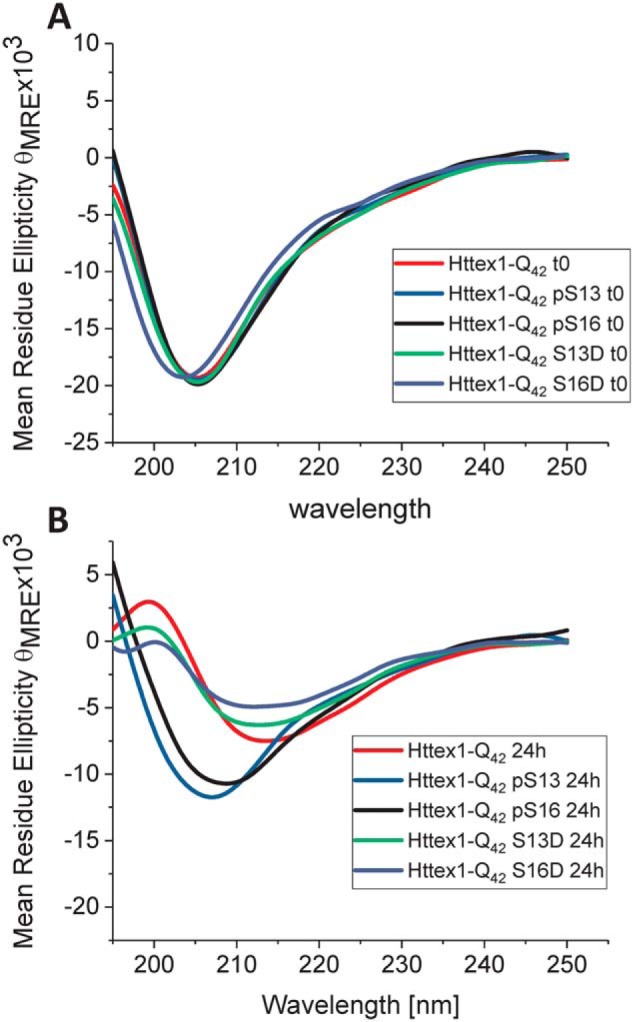
**Only unmodified Httex1 and the S13D and S16D mutants are able to undergo a transition from random coil to β-sheet structure upon aggregation.** Shown are CD spectra of singly phosphorylated (at Ser-13 or Ser-16) mutant Httex1 and the corresponding phosphomimetic proteins at 0 h (*A*) and 24 h (*B*).

By CD spectroscopy, the unmodified Httex1-Q_42_ protein underwent a shift in secondary structure that was consistent with a transition from unstructured (λ_min_ = 205 nm) to predominantly β-sheet structure (λ_min_ = 212–215 nm) within 24 h (Fig. 4). For proteins bearing single phosphomimetic mutations (S13D Httex1-Q_42_ and S16D Httex1-Q_42_), the CD spectra at 24 h were indistinguishable from those of the unmodified Httex1-Q_42_ (Fig. 4*B*)_,_ consistent with the nearly complete loss of soluble protein by sedimentation analysis. In contrast, CD analysis of proteins bearing a single phosphorylation (pSer-13 Httex1-Q_42_ and pSer-16 Httex1-Q_42_) at this time point showed only a decrease in the CD signal without a significant shift in λ_min_.

**Figure 5.**
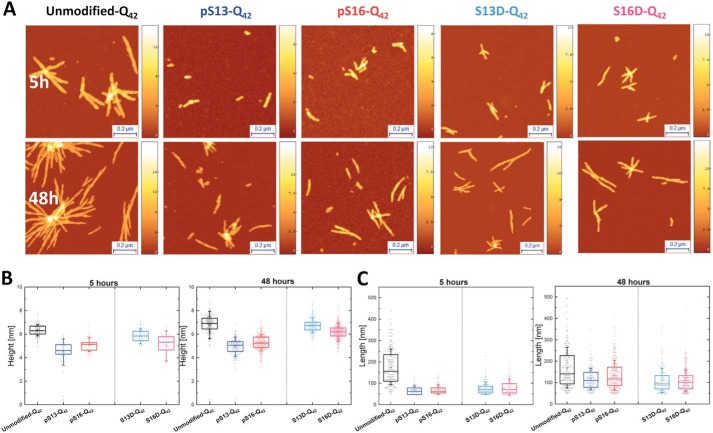
**Phosphorylation at Ser-13 or Ser-16 results in the formation of fibrils with significantly reduced heights compared with the unmodified Httex1-Q_42_ and the corresponding phosphomimetic mutants.** *A*, AFM images of aggregates formed by mutant 42Q Httex1 Q18A phosphorylated at Ser-13 or Ser-16 and the corresponding phosphomimetic mutants. *B* and *C*, quantitative analysis of the height (*B*) and length (*C*) of these aggregates measured from AFM images (*z scale bars* are in nm). All scatter plots of height differ with a statistical significance of at least *p* < 0.05. All scatter plots of the length of the modified aggregates differ from the unmodified with a statistical significance of at least *p* < 0.05. *Error bars*, S.D.

To determine the effect of phosphorylation on the morphological properties of mutant Httex1 fibrils, we performed atomic force microscopy (AFM) high-resolution imaging and statistical analysis on the aggregates formed by each protein. Consistent with the *in vitro* aggregation data, we observed that the phosphorylated proteins pSer-13-Q_42_ and pSer-16-Q_42_ formed significantly fewer aggregates compared with the unmodified Q_42_. However, the data presented in Fig. 5 focus on the quantitative comparison of the fibril morphology, as determined by assessing changes in the height and length distribution of the aggregates formed by each Httex1 variant. The AFM images of the unmodified Httex1-Q_42_ at as early as 5 h of incubation revealed mature fibrillar aggregates with average heights of 6.3 ± 0.5 nm and average lengths of 176 ± 83 nm (Fig. 5). The phosphorylated proteins, pSer-13 and pSer-16-Httex1-Q_42_, produced significantly smaller aggregates with average heights of 4.7 ± 0.6 and 5.1 ± 0.6 nm and average lengths of 67 ± 20 and 70 ± 22 nm, respectively. Only the aggregate length, and not the height of these structures, was observed to increase after 48 h. Interestingly, mutant Httex1 protein bearing a single phosphomimetic mutation (S13D Httex1-Q_42_ and S16D Httex1-Q_42_) formed aggregates at 5 h with average heights of 5.8 ± 0.6 and 5.2 ± 0.8 nm and average lengths of 77 ± 29 and 82 ± 38 nm, respectively (Fig. 5, *B* and *C*). After 48 h, the height of the Ser → Asp mutant Httex1 aggregates had increased notably to sizes comparable with unmodified Httex1-Q_42_, suggesting that the introduction of phosphomimetic mutation at a single serine residue only slightly influences the final morphology of the fibrils, consistent with the ability of the WT, S13D, and S16D Httex1-Q_42_ to form β-sheet–rich aggregates (Fig. 4) (51). In contrast, phosphorylation at Ser-13 or Ser-16 resulted in fibrils with significantly reduced heights compared with the unmodified Httex1-Q_42_. Interestingly, Httex1-Q_42_ bearing *bona fide* phosphorylation or phosphomimetic mutations at Ser-13 or Ser-16 exhibited similar fibril length distributions after 48 h. Together, these findings combined with the CD data suggest that phosphorylation at Ser-13 or Ser-16 residues significantly reduces the rate of fibril formation and inhibits transition to a β-sheet–rich conformation, but does not alter the overall length distribution of the Httex1 fibrils. In contrast, single Ser → Asp substitution at these residues does not inhibit the aggregation of Httex1-Q_42_ or alter the ability of the protein to form β-sheet–rich aggregates.

### Diphosphorylation at Ser-13/Ser-16 inhibits mutant Httex1 aggregation

Phosphorylation at both Ser-13 and Ser-16 was shown to play important roles in modulating Htt pathology in animal models of HD (31). Different studies using phosphomimetic mutations (S13D/S16D) or Httex1-like peptides showed that phosphorylation at both residues (pSer-13/pSer-16) influence the aggregation of Httex1 and reverses Htt's pathology in an HD mouse model (23, 31, 32). However, chemical and enzymatic tools were not available to determine the structural consequences of the Ser-13/Ser-16 phosphorylation on full-length and untagged Httex1 aggregation. Therefore, we sought to use our semisynthetic strategy to produce mutant Httex1 bearing *bona fide* phosphorylation of both of these residues (pSer-13/pSer-16), and to compare its aggregation properties with the commonly used corresponding phosphomimetic mutant (S13D/S16D).

**Figure 6.**
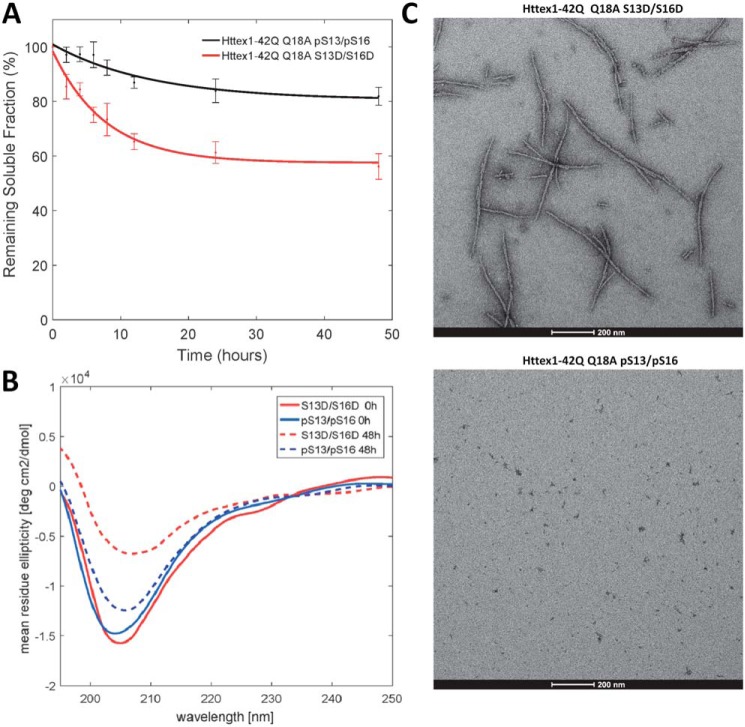
**Phosphorylation at both Ser-13 and Ser-16 inhibits the aggregation of Httex1-Q_42_ Q18A, whereas the S13D/S16D Httex1-Q_42_ Q18A exhibits a marked reduction in aggregation but retains the ability to form fibrils.** *A*, comparative analysis of the aggregation of diphosphorylated Q18A (pSer-13/pSer-16) and diphosphomimetic (S13D/S16D) mutant (42Q) Httex1 at 2.5 μm using the UHPLC sedimentation assay (*error bars*, S.D. derived from *n* = 3 independent experiments). *B*, CD analysis of the samples at 0 and after 48 h of incubation at room temperature. *C*, TEM images of the final aggregation time point (48 h). *Scale bars*, 200 nm.

As shown in Fig. 6*A*, phosphorylation of both Ser-13 and Ser-16, pSer-13/pSer-16 Httex1-Q_42_, resulted in a dramatic inhibition of Httex1–43Q aggregation as determined by both the sedimentation assay and transmission EM (TEM) (Fig. 6, *A* and *C*). The corresponding phosphomimetic mutant (S13D/S16D Httex1-Q_42_) exhibited significantly slower aggregation compared with the unmodified protein or the single phosphomimetic mutants (S13D or S16D; Fig. 3), which individually did not have any effect on mutant Httex1 aggregation. After 48 h of incubation, 82% of the pSer-13/pSer-16 Httex1-Q_42_ protein remained soluble compared with 60% for S13D/S16D Httex1-Q_42_ (Fig. 6*A*). These observations were confirmed by CD spectroscopy, which revealed no significant reduction or shift in CD spectra of pSer-13/pSer-16 Httex1-Q_42_ after 48 h (Fig. 6*B*), suggesting that the protein exists predominantly in monomeric and/or nonstructured low-molecular weight oligomeric forms. In the case of the disphosphomimetic protein (S13D/S16D Httex1-Q_42_) mutant, a significant reduction in the CD signal was observed (Fig. 6*B*), consistent with the loss of soluble protein due to the increased aggregation propensity of this protein and detection of fibrils by TEM (Fig. 6*C*). These results demonstrate that phosphorylation at both serine residues (pSer-13/pSer-16 Httex1-Q_42_) results in a strong inhibitory effect on mutant Httex1 aggregation and that the introduction of phosphomimetic mutations at both of these residues also results in significant inhibition of mutant Httex1 aggregation, but does not fully reproduce the effect of *bona fide* phosphorylation.

### Serine phosphorylation at Ser-13 and/or Ser-16 inhibits WT Httex1 aggregation

**Figure 7.**
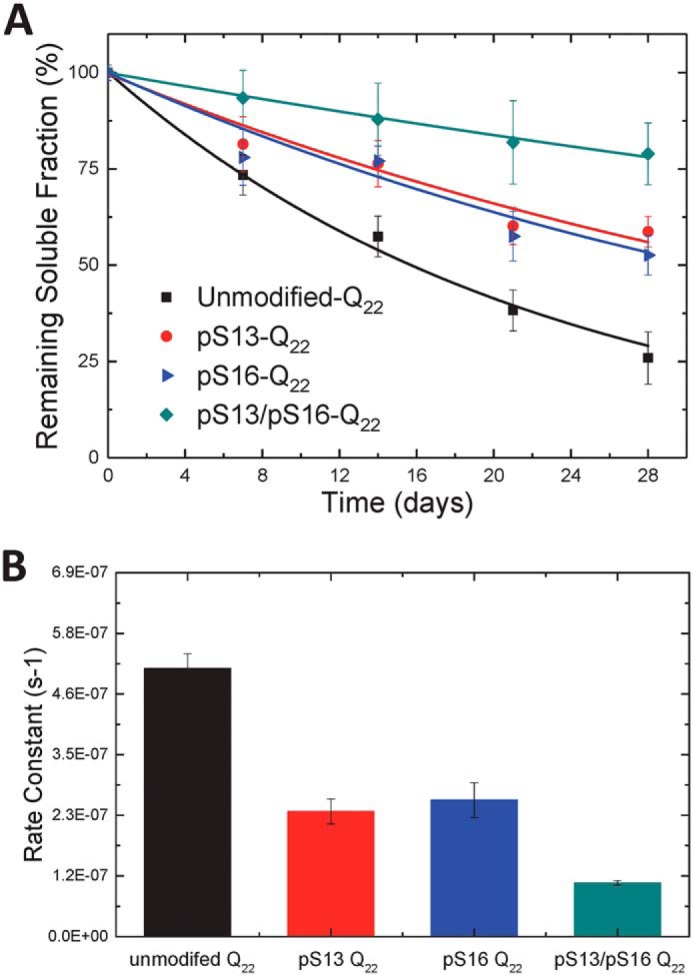
**Serine phosphorylation at Ser-13 and/or Ser-16 inhibits WT Httex1 aggregation.** *A*, comparison of the aggregation of unmodified, singly phosphorylated, and diphosphorylated WT (22Q) Q18A Httex1 protein at 5 μm as determined using the UHPLC sedimentation assay (*error bars*, S.D. derived from *n* = 10 for unmodified, *n* = 6 for pSer-13, *n* = 5 for pSer-16, and *n* = 8 for pSer-13/pSer-16). *B*, calculated aggregation rates.

Previous reports from our group have shown that Httex1 proteins with polyQ repeat lengths well below the pathological threshold (23Q) are able to aggregate and form oligomers and fibrils *in vitro*, albeit at a much lower rate, over a long period of time (days) and to a lesser extent compared with mutant forms of Httex1 with polyQ repeats of >38Q (26, 42). To evaluate the effect of serine phosphorylation on the biophysical properties of WT Httex1, the aggregation of unmodified Httex1-Q_22_, pSer-13 Httex1-Q_22_, pSer-16 Httex1-Q_22_, and pSer-13/pSer-16 Httex1-Q_22_ Httex1 was monitored using the *in vitro* sedimentation assay described above (26). Fig. 7*A* shows that serine phosphorylation at Ser-13 or Ser-16 reduced the rate and extent of WT Httex1 aggregation. For unmodified Httex1-Q_22_ Httex1, 75% of the protein aggregated after 4 weeks of incubation at 37 °C. In contrast, in the case of pSer-13 Httex1-Q_22_ or pSer-16 Httex1-Q_22_ Httex1, ∼40% of the protein was aggregated, indicating that phosphorylation at either residue reduced the extent of Httex1 aggregation to a similar degree. A much stronger effect was observed when both Ser-13 and Ser-16 residues were phosphorylated (pSer-13/pSer-16 Httex1-Q_22_), where <20% of the protein was aggregated after 4 weeks. The amount of soluble protein (monomers and nonsedimentable oligomers) remaining at each intermediate time point was always greater for the phosphorylated Httex1 proteins, indicating a reduction of the aggregation rate as well. As in the case of Q_42_ proteins (see above), the approximate relative aggregation rates were calculated from the sedimentation data. The rates (ks^−1^) were found to be ∼50% for pSer-13 Httex1-Q_22_ and pSer-16 Httex1-Q_22_ Httex1 and ∼20% for pSer-13/pSer-16 Httex1-Q_22_ Httex1 (Fig. 7*B*) relative to the rate of unmodified Httex1-Q_22_ Httex1 protein. The inhibitory effect of serine phosphorylation on WT Httex1 aggregation appeared to be additive, with the diphosphorylated Httex1 exhibiting approximately twice the effect of single phosphorylation at Ser-13 or Ser-16.

### Serine phosphorylation disrupts the amphipathic α-helix of the Htt Nt17 domain

To determine the effect of phosphorylation on the conformational properties of Nt17, we assessed the effect of phosphorylation at Ser-13 and/or Ser-16 on the helix propensity of Nt17 in buffered solutions or upon binding to membranes. The transient helical nature of Nt17 combined with the fact that the vast majority of the Httex1 sequence beyond Nt17 exists in a disordered conformation dominates the CD spectra of the protein, thus making it very difficult to discern secondary structural changes within the Nt17 domain by CD spectroscopy. Therefore, we used synthetic N-terminal peptides bearing the first 19 amino acids of Httex1 (MATLEKLMKAFESLKSFQQ) and where Ser-13, Ser-16, or both residues were phosphorylated.

**Figure 8.**
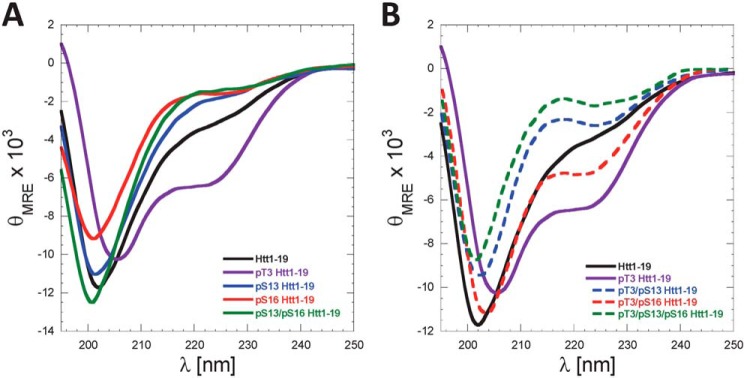
**Serine phosphorylation at Ser-13 and/or Ser-16 disrupts the amphipathic α-helix of Nt17 and reverses pThr-3–induced α-helix formation of Nt17.** *A*, far-UV CD spectra (θ_MRE_ = mean residue ellipticity) of Htt1–19 peptides bearing single (pThr-3, pSer-13, or pSer-16) or multiple phosphorylated (pSer-13/pSer-16) residues at 60 μm (pH 7.4). *A*, phosphorylation of Thr-3 increases the helicity of Nt17, whereas phosphorylation at Ser-13, Ser-16, or both residues reduces the helical propensity of Nt17. *B*, phosphorylation at Ser-13, Ser-16, or both residues disrupts pThr-3–induced α-helix formation of Nt17. To allow for better visualization and comparison of the data and the effect of phosphorylation at Ser-13 and/or Ser-16 on pThr-3–induced α-helix formation, the far-UV CD spectra of Htt 1–19 and Htt 1–19 pThr-3 were replotted again in *B*.

The Nt17 exists predominantly in a disordered conformation but exhibits a high propensity to form transient helical conformations. This is consistent with their CD spectra, which show a minimum at 200–205 nm and small shoulder at 218–225 nm (Fig. 8*A*). Importantly, serine phosphorylation at Ser-13 and/or Ser-16 resulted in a decrease in α-helical content as indicated by the reduction of this shoulder minimum compared with the unmodified Nt19 peptide (Fig. 8*A*). These findings are consistent with previous reports using Httex1-like model peptides containing the Nt17 peptide (32). Recently, we showed that Thr-3 phosphorylation results in significant stabilization of the α-helical conformation of Nt17 and a significant increase in Nt17 α-helical contents measured by CD spectroscopy and NMR (24). To determine whether phosphorylation at Ser-13 and/or Ser-16 could disrupt the stable helical conformation of Nt17 induced by phosphorylation at Thr-3, we synthesized peptides that are phosphorylated at Thr-3 and Ser-13, Thr-3 and Ser-16, and Thr-3 and both Ser-13 and Ser-16 and assessed their conformation by CD. Phosphorylation at Ser-13 resulted in a stronger destabilization of the pThr-3–induced α-helix formation compared with phosphorylation at Ser-16 (Fig. 8*B*). Phosphorylation of all residues (Thr-3, Ser-13, and Ser-16) resulted in complete disruption of the Nt19 helical conformation. Indeed, the triphosphorylated peptide showed a CD spectrum that exhibits even less α-helical content than the unmodified peptide, indicating that phosphorylation at both Ser-13 and Ser-16 completely reverses the effect of Thr-3 phosphorylation on the secondary structure of Nt17 (Fig. 8*B*). Taken together, these results reveal a novel phosphorylation-dependent conformational switch whereby the α-helical conformation of Nt17 is regulated by phosphorylation/dephosphorylation of Thr-3 or by cross-talk between Thr-3 and Ser-13 and/or Ser-16 phosphorylation.

**Figure 9.**
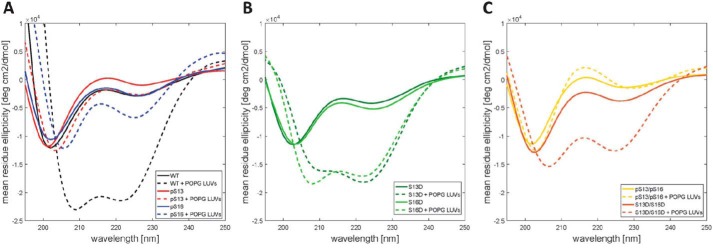
**Phosphorylation at Ser-13 and/or Ser-16, but not the phosphomimetic (S13D and/or S16D) mutations, inhibits helix formation and binding of the Nt17 peptides to LUVs.** *A*, far-UV CD spectra of WT, pSer-13, and pSer-16 Nt17 in the presence and absence of LUVs. *B*, far-UV CD spectra of S13D and S16D in the presence and absence of LUVs. *C*, far-UV CD spectra of the diphosphorylated (pSer-13/pSer-16) Nt17 and the corresponding phosphomimetic mutant(S13D/S16D) in the presence and absence of LUVs.

The Nt17 has been shown to adopt α-helical structure upon membrane binding (52), acts as a membrane anchor for Httex1, and mediates its aggregation on membranes (53). To determine the effect of phosphorylation on Nt17 membrane-binding properties, we assessed the effect of the phosphorylation at Ser-13 and/or Ser-16 and their corresponding phosphomimetic variants on the binding of the Nt17 peptide to 1-palmitoyl-2-oleoyl-*sn*-glycero-3-phospho-(1′-racglycerol) (POPG) as a membrane model. POPG large unilamellar vesicles (LUVs) with an average diameter of 100 nm were used at 5 molar eq. As expected, the unmodified Nt17 peptide formed an α-helix upon incubation with LUVs (Fig. 9*A*). Phosphorylation at Ser-13 or Ser-16 significantly inhibited the random coil–to–α-helix transition by Nt17 in the presence of lipid vesicles, with phosphorylation at Ser-13 inducing the strongest effect. When both Ser-13 and Ser-16 residues were phosphorylated (pSer-13/pSer-16), the Nt17 peptide failed to adopt α-helical conformations in the presence of membranes and exhibited CD spectra that are similar to those of Nt17 in the absence of membranes (Fig. 9, *A* and *C*). In contrast, the introduction of the phosphomimetic mutation (Ser → Asp) at Ser-13 or Ser-16 or both residues did not influence the ability of Nt17 to adopt an α-helical conformation in the presence of POPG or 1,2-dimyristoyl-*sn*-glycero-3-phosphocholine LUVs (Fig. 9 (*B* and *C*) and Fig. S1). These results demonstrate that the phosphomimetics do not reproduce the effect of phosphorylation on Nt17 conformation upon membrane binding, highlighting the importance of using *bona fide* phosphorylated proteins/peptides to study the effect of this modification on the structural properties of Httex1.

### Serine phosphorylation prompts the internalization and subcellular targeting of Httex1 in neurons

**Figure 10.**
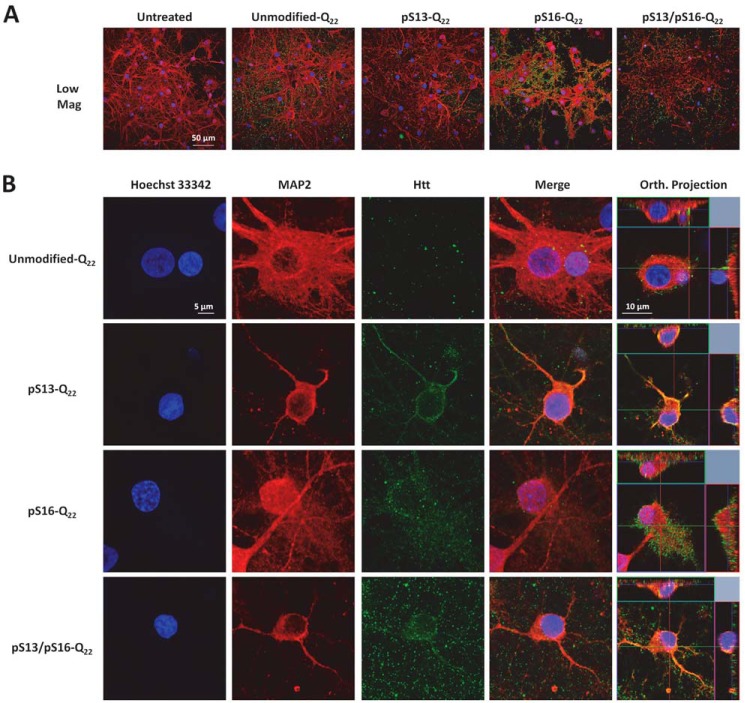
**Effect of phosphorylation at Ser-13 and/or Ser-16 on the internalization of WT (22Q) Httex1 PFFs in striatal neurons.** Primary rat striatal neurons treated with 0.5 μm unmodified or PTM Q_22_ PFFs were immunostained using the MW8 anti-total Htt antibody. Staining for MAP2 and Hoechst-33342 was also performed to reveal neuronal cell bodies and nuclei, respectively. *A*, low magnification imaging showing that the MW8 antibody equivalently detected unmodified and PTM Q_22_ Httex1 PFFs, in contrast to the untreated condition. *B*, high magnification confocal imaging of individual neurons showing that while unmodified Q_22_ PFFs are mostly not internalized, some neurons treated with pSer-13-Q_22_, pSer-16-Q_22_, and pSer-13/pSer-16-Q_22_ Httex1 PFFs exhibit prominent internalization of PFFs, as established by orthogonal projection of *z*-stacks (*Orth. Projection*, *rightmost lane*). Orthogonal projections of all imaged channels of the same neurons are presented in Fig. S3*A*.

**Figure 11.**
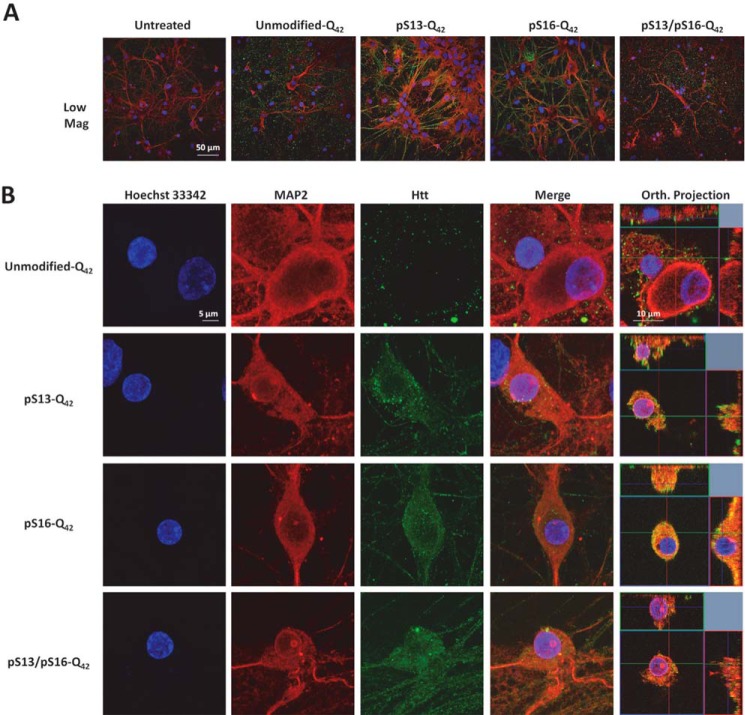
**Effect of phosphorylation at Ser-13 and/or Ser-16 on the internalization of mutant (Q_42_) Httex1 PFFs in striatal neurons.** Primary rat striatal neurons treated with 0.5 μm unmodified or PTM Q_42_ PFFs were immunostained using the MW8 anti-total Htt antibody. MAP2 and Hoechst-33342 staining were also performed to reveal neuronal cell bodies and nuclei, respectively. *A*, low-magnification imaging showing that the MW8 antibody equivalently detected unmodified and PTM Q_42_ PFFs in contrast to the untreated condition. *B*, high-magnification confocal imaging of individual neurons showing that whereas unmodified-Q_42_ PFFs are mostly not internalized, some neurons treated with pSer-13-Q_42_, pSer-16-Q_42_, and pSer-13/pSer-16-Q_42_ Httex1 PFFs exhibit prominent internalization of PFFs, as established by orthogonal projection of *z*-stacks (*Orth. Projection*, *rightmost lane*). Orthogonal projections of all imaged channels of the same neurons are presented in Fig. S3*B*.

Increasing lines of evidence from cellular models (54–57), animal models (58–60), and recently from HD patients (61) support a role for the cell-to-cell transmission and propagation of Htt aggregates in the pathogenesis of HD. Therefore, we sought to investigate the effect of serine phosphorylation on Httex1 aggregate internalization and subcellular targeting in striatal primary neurons. To achieve this goal, we first generated preformed fibrillar species (PFFs) of each of the different proteins by incubating monomers at a high concentration (60–100 μm) at 37 °C for several days. The preparations were then briefly sonicated to generate small fibrillar fragments and then characterized by TEM (Fig. S2*A*). Dot blot analysis (Fig. S2*B*) was also performed to establish that the relative concentrations of the different preparations were similar. Next, we treated primary striatal neurons with 0.5 μm PFFs (Httex1-Q_22_ or Httex1-Q_42_) with single/double phosphoserine residues or with the unmodified PFFs as a control. Notably, immunocytochemistry using the MW8 anti-Httex1 antibody (C-terminal) showed that whereas unmodified PFFs were mostly distributed outside the cellular membrane with very rare instances of internalization of a few foci of PFFs, neurons treated with phosphoserine-bearing Httex1-Q22 or Httex1-Q42 PFFs displayed, in addition to these distributions, uptake of large quantities of PFFs that appeared to fill up the cytoplasm in a homogeneous manner (Figs. 10 and 11). Orthogonal projections of high-resolution confocal *z*-stacks verified the intracellular localization of the phosphoserine aggregates in treated neurons, in contrast to the unmodified PFFs, which mostly appeared to sit on the extracellular membrane (Figs. 10 and 11 and Fig. S3).

**Figure 12.**
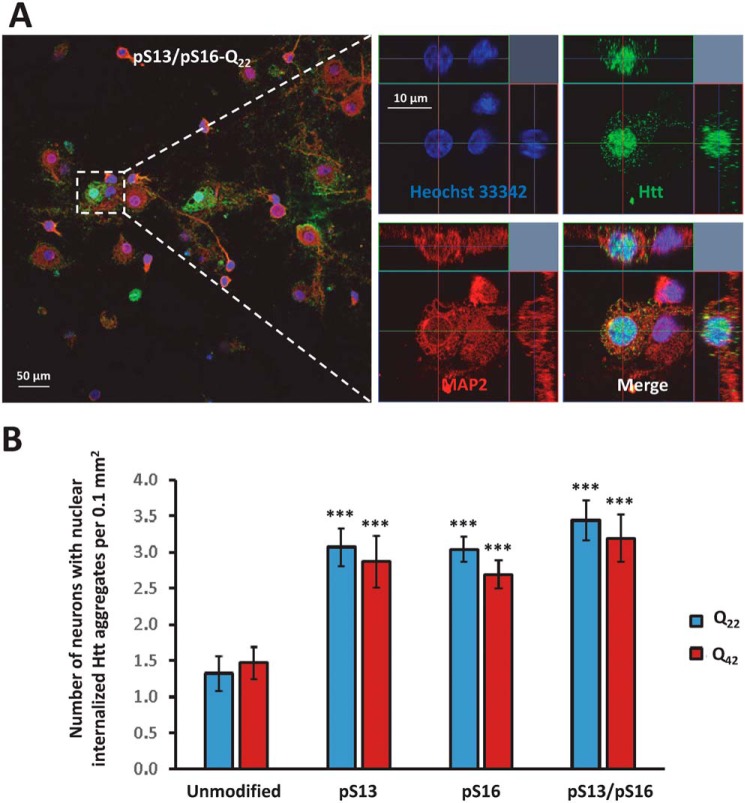
**Effect of phosphorylation at Ser-13 and/or Ser-16 on the subcellular localization of mutant (Q_42_) PFFs in striatal neurons.** *A*, immunostaining using the MW8 antibody revealing nuclear localization of internalized pSer-13/pSer-16-Q_22_ PFFs in a proportion of neurons. A low-magnification view is shown in the *left panel*, and the *right panel* shows high-resolution images of an individual neuron exhibiting nuclear localization. *B*, significantly more neurons treated with phosphorylated Q_22_ and Q_42_ Httex1 PFFs exhibit nuclear localization per 0.1 mm^2^ compared with the controls treated with unmodified Q_22_ or Q_42_ Httex1 PFFs. The *asterisks* denote *p* < 0.001 determined by one-way analysis of variance and Scheffé post hoc statistical analysis. *Error bars*, S.D.

In addition, some of the neurons that had internalized PFFs bearing serine phosphorylation also exhibited additional subcellular targeting to the nuclear compartment (Fig. 12*A*). To dissect the effect of single *versus* double phosphorylation on the nuclear targeting of Httex1 aggregates, we quantified the number of neurons exhibiting nuclear Httex1 localization upon treatment with unmodified *versus* the different phosphoserine-bearing PFF variants. Notably, we found that both single and double phosphorylation of Nt17 significantly enhanced the targeting Httex1-Q_22_ and Httex1-Q_42_ PFFs to the nuclear compartment compared with unmodified PFFs (Fig. 12*B*), with neurons treated with pSer-13/pSer-16 double-phosphorylated PFFs exhibiting a slight tendency toward increased nuclear localization. Nevertheless, no statistically significant differences were noted among the three phosphorylated variants, indicating that a single phosphorylation event at either Ser-13 or Ser-16 is sufficient to prompt the nuclear enrichment of Httex1-Q_22_ or Httex1-Q_42_ aggregates.

## Discussion

Protein phosphorylation is a dynamic PTM that allows precise temporal control of protein structure, function, and subcellular localization. Phosphorylation of proteins implicated in neurodegenerative diseases such as Tau in Alzheimer's disease, α-synuclein in Parkinson's disease, and TDP-43 in ALS, seems to correlate with the formation of pathological aggregates and disease progression (62). In Huntington's disease, phosphorylation of N-terminal serine and threonine residues has been detected in human brains, transgenic models, and cell culture models of HD (5, 31, 63, 64). However, the role of phosphorylation in regulating the structure, aggregation, and HD pathology remains poorly understood, primarily due to the lack of tools that enable site-specific and efficient modulation of phosphorylation *in vitro* and *in vivo*.

The observation that phosphomimetic mutations at serines 13 and 16 attenuate disease progression and accumulation of Htt aggregates in the BACHD mouse model highlights the potential role of phosphorylation in regulating Htt function in health and disease and underscores the critical importance of achieving a more in-depth understanding of the effects of phosphorylation at these residues (31). The knowledge gained from such studies could have important implications for the development of novel therapies and diagnostic strategies based on modulating and/or assessing the levels of phosphorylation at these residues (63). Toward these goals, we utilized protein semisynthetic strategies that were recently developed by our group to generate WT and expanded forms of exon 1 of the huntingtin protein phosphorylated at Ser-13, Ser-16, or both residues. To allow comparison and correlation of our findings with previously published studies that relied mainly on the use of phosphomimetic mutations, we generated the corresponding phosphomimetic mutant Httex1: S13D, S16D, and S13D/S16D. In all cases, we assessed and compared the structure, membrane binding, and aggregation properties of each protein with the corresponding unmodified proteins using multiple assays, including sedimentation assays, AFM, TEM, and CD spectroscopy. Moreover, we investigated, for the first time, the effect of serine phosphorylation on the internalization and subcellular targeting of Httex1 aggregates in primary striatal cultures.

Previously, biophysical analysis of the effects of N-terminal serine phosphorylation on the *in vitro* aggregation properties of Httex1 utilized peptide model systems in which the last 40 C-terminal residues of the Httex1 protein were deleted and/or replaced by a solubilizing lysine repeat domain (23, 31). Despite these limitations, the results of the aggregation studies using this truncated peptide model are consistent with our observations using tag-free semisynthetic Httex1 proteins and show that serine phosphorylation at either or both Ser-13 and Ser-16 reduces the aggregation rate of the WT and mutant Httex1, with diphosphorylation having the strongest inhibitory effect. However, in all previous studies, a direct comparison of the effect of phosphorylation and phosphomimetic mutations on the structural properties of Httex1 or Nt17 peptides was never carried out. We postulated that conducting such comparative studies is crucial to determine to what extent the phosphomimetic mutations reproduce all of the effects of phosphorylation. Our results demonstrated that phosphorylation at Ser-13 or Ser-16 significantly reduced the rate and extent of WT and mutant Httex1 aggregation. Substitution of a single serine by aspartate to mimic phosphorylation failed to reproduce the inhibitory effect of single phosphorylation in the context of mutant Httex1. Previous studies by Wetzel and colleagues (23) only compared the effect of *bona fide* phosphorylation at Ser-13 and/or Ser-16 with the diphosphomimetics form of mutant Httex1 and showed that the latter exhibits aggregation kinetics similar to those of the singly phosphorylated proteins (pSer-13 or pSer-16) in their Httex1 model system (Nt17-Q37-P10-K2). Our results suggest that single phosphomimetic mutations are not sufficient to reproduce the effect of phosphorylation at single serine residues within Nt17 of mutant Httex1. Phosphorylation of both residues (pSer-13/pSer-16) resulted in a marked decrease in the rate of WT and mutant Httex1 aggregation and fibril formation. Whereas the S13D/S16D double mutant was able to partially reproduce the inhibitory effect of diphosphorylation pSer-13/pSer-16 on the aggregation of mutant Httex1-Q_42_, it did not reproduce the effect of phosphorylation on the propensity of Httex1-Q_42_ to form β-sheet–rich aggregate or the effect of phosphorylation on the membrane-binding properties of the Nt17 peptide. These results and previous findings by Wetzel and colleagues (23) are consistent with the aggregation-inhibitory effects being dominated by charge-mediated mechanisms. The same charge effect was suggested to play a role in Nt17 lipid binding, where it has been shown that acetylation at the three lysine residues decreased the binding of Httex1-like peptide model to lipids. However, herein we show that the effects of phosphorylation on membrane binding are strongly dependent on the effect of phosphorylation on the conformation of the Nt17 peptide. Our CD studies showed that phosphorylation at Ser-13 and/or Ser-16 led to complete disruption of Nt17 helix formation in the absence or presence of lipid vesicles and reversed the pThr-3–induced α-helix formation of Nt17. These findings are consistent with a recent study by Daldin *et al.* (65) demonstrating that phosphorylation of Ser-13 and Ser-16 (pSer-13/pSer-16-Q_42_) conveys increased conformational flexibility in temperature and polyQ repeat length–dependent experiments. They proposed that disruption of the Nt17 α-helix is responsible for this observation. Further studies using NMR and other high-resolution techniques are needed to determine the mechanisms by which phosphorylation-dependent modulation of the conformation of Httex1 contributes to the strong inhibitory effect of this PTM on mutant Httex1 aggregation.

Additionally, serine modifications (pSer-13, pSer-16, pSer-13/pSer-16, and S13D/S16D) in the context of the Httex1 peptide model were reported to result in the formation of both filamentous and amorphous aggregate species by TEM (23). However, comparable structures were not observed for any of the Httex1 protein in our studies. In contrast, AFM analyses of the aggregates generated by mutant pSer-13, pSer-16, S13D, S16D, and S13D/S16D Httex1 proteins showed aggregates of similar morphology to unmodified Httex1 but also revealed differences in the dimensions of these aggregates. It is plausible that these differences in aggregation morphology between the two studies could be caused by the removal of a significant part of the C terminus, which is known to play an important role in enhancing the solubility of Httex1, or by the presence of the nonnative lysine residue in the Httex1 model peptide.

A recent study by Branco-Santos suggested that a single phosphomimic mutation in mutant Httex1 (97Q) is sufficient to completely abolish Httex1 aggregation and inclusion formation (25). These results are not consistent with our observations and previous studies reported by Wetzel and co-workers (23). The discrepancy between their findings and our results could be due to the differences in the aggregation conditions (*in vitro versus* in cells and *in vivo*) or the Httex1 constructs used by both groups. Whereas our studies are based on direct measurements of the aggregation of untagged mutant Httex1–42Q, the studies by Branco-Santos *et al.* (25) relied primarily on indirect assessment of aggregation using a fluorescence biocomplementation assay. Furthermore, the nature of the aggregates (fibrillar or amorphous) formed by these mutant Httex1 fusion proteins remains unknown.

Next, having generated semisynthetic serine-phosphorylated Httex1 proteins, we sought to investigate the effect of *bona fide* phosphorylation on Httex1 aggregate internalization and subcellular targeting in primary striatal neurons. Notably, we found that serine phosphorylation prompted the internalization of aggregated Httex1 and its accumulation within the nuclear compartment. Our results are consistent with a previous study showing that Hek293T cells expressing YFP-Htt-nt17 with phosphomimetic mutations at Ser-13/Ser-16 display enhanced nuclear localization, due to the impaired interaction with the nuclear export protein CRM1 (29). Similar results were observed in the study reported by Thompson *et al.* (6), which also showed that overexpressed GFP-Htt-ex1–97Q with the S13D-S16D mutations exhibits an increased ratio of nuclear to cytoplasmic fluorescence. However, it is noteworthy that in these studies it was not possible to distinguish which species is translocating to the nucleus and therefore the effect of phosphorylation on the secretion and uptake of Httex1 aggregates has not been investigated. Our results demonstrate, for the first time, that genuine phosphorylation of untagged exogenous Httex1 fibrils can prompt the internalization and accumulation of Htt within the nuclear compartment. Moreover, these results suggest that in this case, the phosphomimetics may be reproducing the effect of phosphorylation in directing the subcellular localization of Httex1. These experiments highlight the utility of these protein tools to investigate not only the biophysical consequences of Httex1 phosphorylation but also the interplay between site-selective protein modification, polyQ repeat length, and protein conformation on the biological properties of Httex1 and set the stage and set the stage of further studies to elucidate the role of PTMs in regulating Htt cell-to-cell propagation and pathology spreading.

In conclusion, a detailed analysis of the effects of serine phosphorylation on biophysical properties of Httex1 revealed that phosphorylation at serine 13 and/or 16 provokes a remarkable inhibition of aggregation of WT and mutant Httex1. A comparative analysis of the effects of the phosphomimetic mutations revealed that the introduction of Ser → Asp mutations to mimic phosphorylation at both Ser-13 and Ser-16 significantly reproduces the effect of phosphorylation on Httex1 aggregation, but is insufficient to reproduce the effect of phosphorylation on the conformation of Nt17 and its ability to bind membranes. In primary neurons, phosphorylation of Httex1 aggregates enhances their internalization and nuclear localization. In addition, our studies revealed a novel phosphorylation-dependent molecular switch, involving cross-talk between phosphorylation at Thr-3 and Ser-13 and/or -16, for regulating the conformation of the N terminus of Htt. Given that phosphorylation at these residues has been implicated in regulating several cellular and toxic properties of Htt, deciphering the PTM-dependent interactome of Htt and unraveling the network of kinases and phosphatases involved in regulating phosphorylation at Ser-13 and Ser-16 should be a top priority to advance our understanding of Htt biology in health and disease. This will facilitate the development of novel strategies to protect against Httex1 aggregation and slow the progression of HD based on selective modulation of Nt17 phosphorylation and regulation of PTM-dependent Httex1 interactions with other proteins, ligands, and cellular compartments.

## Experimental procedures

### Materials

The peptides used in these studies were synthesized by Integrated Research Biotech Model (IRBM) in collaboration with the CHDI Foundation or purchased from CSBio Co. Glacial acetic acid, TFA, guanidine hydrochloride (GdnHCl), tris(2-carboxyethyl)phosphine (TCEP), methoxyaminehydrochloride (MeONH_4_Cl), MPAA, 2-methyl-2-propanethiol, sodium borohydride (NaBH_4_), nickel acetate, l-proline, l-methionine, and d-trehalose were purchased from Sigma-Aldrich. The 2–2′-azobis[2-(2-imidazolin-2-yl)propane] dihydrochloride (VA-044) was purchased from Wako. HPLC-grade acetonitrile was purchased from Macherey-Nagel. ER2566 *Escherichia coli* (E6901S)-competent cells were purchased from New England Biolabs. Phenylmethanesulfonyl fluoride (PMSF) was purchased from Axonlab. Urea, DMSO, and isopropyl β-d-thiogalactopyranoside were purchased from Applichem. CLAP protease inhibitor (1000×) consisted of 2.5 mg/ml chymostatin, leupeptin, antipain, and pepstatin A from Applichem dissolved in DMSO. The primary mouse anti-Huntingtin mAb was purchased from Millipore (MAB5492). Secondary goat anti-mouse antibody labeled with Alexa Fluor 680 was purchased from Invitrogen (A-21057). PageRuler Prestained Protein Ladder (26617) from Thermo Scientific was used for SDS-PAGE. Microcon centrifugal filters with a MWCO of 100,000 kDa were obtained from Millipore (MRCF0R100). Formvar carbon film on 200-mesh copper grids (FCF200-Cu) and uranyl formate (16984-59-1) from Electron Microscopy Sciences were used for sample preparation for negative-stain TEM. LC-ESI-MS was performed using a C3 column (Agilent Poroshell 300SB-C3, 1 × 75 mm, 5 μm) with a gradient of 5–95% acetonitrile (0.1% (v/v) formic acid) at 0.3 ml/min over 6 min with UV detection at 214 nm and MS detection on a Thermo LTQ. MALDI-TOF MS was performed on an AB Sciex 4700 instrument using a sinapinic acid matrix. Ultraperformance LC (UPLC) analysis was performed on a Waters UPLC system (Waters Acquity H-Class) using a C8 column (Waters Acquity UPLC BEH300, 1.7 μm, 300 Å, 2.1 × 150 mm) with UV detection at 214 nm using a 2.75-min gradient of 10–90% acetonitrile (0.1% (v/v) TFA) at 0.6 ml/min. Preparatory HPLC was performed on a Waters HPLC system (Waters 2535) using a C4 column (Phenomenex Jupiter C4, 10 μm, 300 Å, 21.2 × 250 mm) with UV detection (Waters 2489) at 214 nm and manual fraction collection.

### Expression and purification of Q18C 18–90 (22Q/42Q) Httex1 protein fragments

Expression of the previously described constructs was performed in *E. coli* BER2566 cells (26). The cells were grown at 37 °C until an *A*_600_ of 0.1 was reached, at which point the incubator was cooled to 14 °C. Expression was then induced at this temperature at an *A*_600_ of 0.45 with 1 mm (22Q) or 0.4 mm (42Q) isopropyl β-d-thiogalactopyranoside. Bacteria were harvested by centrifugation and lysed by ultrasonication in 22Q lysis buffer (40 mm Tris acetate, 5 mm EDTA, 30 mm cysteine, pH 8.3, 1× CLAP, and 0.3 mm PMSF) or the 42Q lysis buffer (4.0 m urea, 40 mm Tris acetate, 5 mm EDTA, 30 mm cysteine, pH 8.3, 1× CLAP, and 0.3 mm PMSF). The supernatant was separated from the cell debris by centrifugation (23,000 × *g*, 2 × 20 min, 4 °C). For 22Q protein, splicing of the Ssp intein was then induced by adjusting the pH to 7.0, and the resulting solution was incubated at room temperature for 3 h. At this point, or directly after separation from the cell debris for 42Q protein, the supernatant was acidified by the addition of 1% TFA and incubated in boiling water for 5 min. The soluble supernatant was separated from the insoluble debris by centrifugation (5000 × *g*, 15 min, 4 °C). After the pH was adjusted to 2.0, the supernatant was filtered (0.2-μm PTFE filter) and purified by preparative HPLC with a gradient from 10 to 40% acetonitrile in H_2_O with 0.1% TFA over 50 min after a 10-min equilibration with 5% acetonitrile. Fractions containing the desired product (elution ∼40 min) were identified by ESI-MS and analyzed for purity by C8-UPLC. Pure fractions were pooled and frozen in liquid nitrogen, and the solvent was removed by lyophilization to yield a white lyophilizate (∼4.0 mg/liter expression for 22Q protein, ∼1.5 mg/liter expression of 42Q protein).

### Semisynthesis and desulfurization of WT (22Q) Httex1 proteins

Recombinant C-terminal Q18C 18–90 (22Q) was first disaggregated by treatment with undiluted TFA (∼200 μl/mg) for 30 min. TFA was removed under a stream of inert gas (N_2_ or argon) to yield a thin film. The film was then resuspended by vortexing in ligation buffer solution (6 m GdnHCl, 50 mm Tris, 50 mm TCEP, pH 7.0) at ∼4 mg/ml. Methoxyamine was then added (100 mm MeONH_4_Cl), the pH was adjusted to 4.0, and the resulting solution was incubated at 37 °C for 1 h (this treatment was forgone for ligations with peptides containing Ser-16 phosphorylation). Next, MPAA (30 mm) was added to the solution, and the pH was adjusted to 6.6 or 7.2 (pH 6.6 was used for ligations with peptides containing Ser-16 phosphorylation). The corresponding N-terminal NBz peptide fragment (1.5 eq) was added to the solution and vortexed, and the pH was rechecked. The reaction was monitored by LC-MS analysis. Upon consumption of the C-terminal Q18C Httex1 18–90 fragment (2–3 h), the solution was diluted in 6 m GdnHCl (to 18 ml), filtered (0.2-μm PTFE filter), and purified by preparative HPLC with a gradient from 20 to 55% acetonitrile in H_2_O, 0.1% TFA over 50 min after a 10-min equilibration with 5% acetonitrile. Fractions containing the desired products (elution ∼35 min) were identified by ESI-MS and analyzed for purity by C8-UPLC. Pure fractions were pooled and frozen in liquid nitrogen, and the solvent was removed by lyophilization to yield a white lyophilizate. The lyophilizate was then disaggregated by treatment with undiluted TFA (∼200 μl/mg) for 30 min. TFA was removed under a stream of inert gas (N_2_ or argon) to yield a thin film. The film was then resuspended by vortexing in the ligation buffer solution (6 m GdnHCl, 50 mm Tris, 500 mm TCEP, pH 7.0) at ∼4 mg/ml. VA-044 and 2-methyl-2-propanethiol were then each added, and the resulting solution was incubated at 37 °C for 1–2 h under argon until LC-MS analysis indicated that desulfurization was completed. The solution was diluted in 6 m GdnHCl (to 18 ml), filtered (0.2-μm PTFE filter), and purified by preparative HPLC with a gradient from 20 to 55% acetonitrile in H_2_O 0.1% TFA over 50 min after a 10-min equilibration at 5% acetonitrile. Fractions containing the desired products (eluted ∼35 min) were identified by ESI-MS and analyzed for purity by C8-UPLC. Pure fractions were pooled and frozen in liquid nitrogen, and the solvent was removed by lyophilization to yield a white lyophylizate (yield of 20–30% over two steps).

### Semisynthesis and desulfurization of WT (42Q) Httex1 proteins

Recombinant C-terminal Q18C 18–90 (42Q) was first disaggregated by treatment with undiluted TFA (∼200 μl/mg) for 30 min. TFA was removed under a stream of inert gas (N_2_ or argon) to yield a thin film. The film was then resuspended by vortexing in the ligation buffer solution (8 m urea, 500 mm l-proline, 30 mm d-trehalose, 50 mm NaH_2_PO_4_, 50 mm TCEP, pH 7.0) at 1 mg/ml. Methoxyamine was then added (100 mm MeONH_4_Cl), the pH was adjusted to 4.0, and the resulting solution was incubated at 37 °C for 1 h (this treatment was forgone for ligations with peptides containing Ser-16 phosphorylation). Then MPAA (30 mm) was then added to the solution, and the pH was adjusted to 6.6 or 7.2 (pH 6.6 was used for ligations with peptides containing Ser-16 phosphorylation). The corresponding N-terminal NBz peptide fragment (1.5 eq) was added to the solution and vortexed, and the pH was confirmed and readjusted if necessary to 6.6 or 7.2. The reaction was monitored by LC-MS analysis. Upon consumption of the C-terminal Q18C Httex1 18–90 fragment (2–3 h), the solution was diluted in 8 m urea (to 18 ml), filtered (0.2-μm PTFE filter), and purified by preparative HPLC with a gradient from 20 to 55% acetonitrile in H_2_O, 0.1% TFA over 50 min after a 10-min equilibration with 5% acetonitrile. Fractions containing the desired products (eluted for ∼35 min) were identified by ESI-MS and analyzed for purity by C8-UPLC. Pure fractions were pooled and frozen in liquid nitrogen, and the solvent was removed by lyophilization to yield a white lyophylizate. The lyophilizate was then disaggregated by treatment with undiluted TFA (∼200 μl/mg) for 30 min. TFA was removed under a stream of inert gas (N_2_ or argon) to yield a thin film. The film was then resuspended by vortexing in the desulfurization buffer solution (20% acetic acid, 30 mm l-methionine, 100 mm TCEP) at 1 mg/ml. Nickel boride suspension was freshly prepared by reduction of nickel acetate as described previously (50) and mixed with the reaction solution (1:4). The solution was incubated at 37 °C with shaking (200 rpm) for 2–3 h or until LC-MS analysis indicated that desulfurization was complete. After, the solution was diluted in 6 m GdnHCl (to 18 ml), filtered (0.2-μm PTFE filter), and purified by preparative HPLC with a gradient from 20 to 55% acetonitrile in H_2_O 0.1% TFA over 50 min after a 10-min equilibration with 5% acetonitrile. Fractions containing the desired products (eluted ∼35 min) were identified by ESI-MS and analyzed for purity by C8-UPLC. Pure fractions were pooled and frozen in liquid nitrogen, and the solvent was removed by lyophilization to yield a white lyophilizate (yield 15–25% over two steps).

### In vitro aggregation analysis of Httex1 proteins

Each protein lyophilizate was weighed into an Eppendorf tube and treated with TFA (15 μl/100 μg) for 30 min for disaggregation. The TFA was removed under a stream of dry nitrogen. The protein was dissolved in 1× PBS (137 mm NaCl, 2.7 mm KCl, 10 mm Na_2_HPO_4_, 2 mm KH_2_PO_4_) to obtain an approximate concentration of 20 μm, and the pH was adjusted to 7.3–7.4 by the addition of a small volume (1–3 μl) of 1 m NaOH. Any remaining preformed aggregates were removed by filtration through molecular weight 100,000 cutoff filters. The protein concentration was then determined using a UPLC calibration curve based on amino acid analysis (detection at λ_214_). The protein was then diluted to the desired concentration (5 or 3 μm for 22Q and 42Q proteins, respectively) with 1× PBS and confirmed by UPLC analysis, and the aggregation experiment was initiated by incubation at the desired temperature (37 and 25 °C for the 22Q and 42Q proteins, respectively). To monitor the soluble protein fraction, the aggregation experiment solution was returned to 25 °C and gently mixed. Next, an aliquot (35 μl) was removed at each indicated time point, and aggregates were removed by centrifugation (20,000 × *g*, 20 min, 4 °C). The supernatant was analyzed by UPLC, and the change in the peak area was used to calculate the fraction remaining compared with *t* = 0. Each protein was analyzed in replicate (*n* = 4–10), and the combined data from these analyses were plotted and fitted to a single-phase exponential decay. To estimate the rate constant of sedimentation and thus aggregation, we used the function, *y* = *Ae*^−^*^b^*^(^*^x^*^)^ + c, where *A* is the initial concentration of soluble protein, *c* is the concentration of soluble protein at infinite time, and *b* is the rate constant. We constrained the values of *A* and *c* to a range varying between 0 and 100 ± the 2% sensitivity error we commit to the measurement for physical reasons. All fits had an adjusted *R*^2^ between 0.97 and 0.99 for Httex1-Q_22_ curves. All Httex1-Q_42_ curves had an adjusted *R*^2^ ranging from 0.93 to 0.98.

### CD analysis

For CD analysis of the aggregation process, aliquots were removed from the aggregation experiments at the indicated time points (100 μl) and analyzed using a Jasco J-815 CD spectrometer in a 1-mm quartz cuvette. The ellipticity was measured from 195 to 250 nm at 25 °C, and the data points were acquired continuously every 0.2 nm at a speed of 50 nm/min with a digital integration time of 2 s and a bandwidth of 1.0 nm. Five spectra of each sample were obtained and averaged. A sample containing buffer only was analyzed and subtracted from each signal. The obtained spectra were smoothed using a binomial filter with a convolution width of 99 data points, and the resulting spectra were plotted as the mean residue molar ellipticity (θ_MRE_).

### TEM

Samples were deposited on Formvar/carbon-coated 200-mesh copper grids (Electron Microscopy Sciences) for 1 min at room temperature, and then grids were washed and stained with 0.75% (w/v) uranyl formate (Electron Microscopy Sciences) solution in water. Prepared grids were imaged using a Tecnai Spirit BioTWIN microscope at 80 kV (LaB6 gun, 0.34 nm line resolution) equipped with a 4k × 4k Eagle CCD camera with a high-sensitivity scintillator from FEI.

### AFM sample preparation, imaging, and statistical analysis

Conventional AFM measurements in air of the samples deposited on positively functionalized mica were performed. To functionalize the surface, after cleaving, the bare mica substrate was incubated with a 10-μl drop of 0.05% (v/v) (3-aminopropyl)triethoxysilane (APTES; Fluka) in Milli-Q water for 1 min at room temperature, rinsed with Milli-Q water, and then dried by the passage of a gentle flow of gaseous nitrogen. Preparation of the mica AFM samples was realized at room temperature by the deposition of a 10-μl aliquot of the fully concentrated solution for 10 min. The sample was then rinsed with ultrapure water and dried with a gentle flow of nitrogen.

High-resolution images (1024 × 1024 pixels) were collected using an NX10 atomic force microscope (Park Systems, Suwon, South Korea) under ambient conditions and in amplitude modulation noncontact (NC-AM) mode. We imaged square areas of 2 × 2 μm^2^ and 4 × 4 μm^2^. We performed all of the measurements using ultra-sharp cantilevers (SSS-NCHR, Park Systems) with a resonance frequency of 330 kHz and typical apical radius of 2 nm. Raw images were flattened with the built-in software (XEI, Park Systems). To maintain consistency in further statistical analysis, all images were processed using the same parameters. First, the images were flattened by a plane and then line by line at a first regression order. This second step was repeated until the flat baseline inline profile of the image was reached. During the process of image flattening, the aggregates were masked from the calculation to avoid a modification and underestimation of their height.

Statistical analysis of the cross-sectional dimensions of the fibrillar structures was performed using a home-built program (DNA-trace). In particular, the software enabled tracing of individual fibrils and the measurement of their length and average height.

### Amino acid analysis

Before each aggregation experiment, 3–5 μg of each protein was dried in an evacuated centrifuge and subjected to amino acid analysis at the Functional Genomic Center Zurich to further confirm that the sample concentration matched the UPLC calibration curve–based calculations.

### Membrane-binding assay

POPG, dimyristoylphosphatidylcholine, or dipalmitoylphosphatidylcholine (Avanti Polar Lipids), were prepared by extrusion as reported previously (66). The phospholipids solution was transferred to a glass vial, and the solvent was evaporated under a stream of nitrogen. The vial was placed in the lyophilizer under vacuum overnight. The lipid film was resuspended by adding PBS buffer to obtain a multilamellar vesicle suspension with a POPG concentration of 1 mg/ml. The lipid solution was subjected to a water bath sonication to disrupt the multilamellar vesicles. LUVs were then prepared by extrusion, applying 21 passes through a polycarbonate filter with a 100-nm average pore diameter (Avestin). Samples for membrane-binding studies were prepared by adding 5 molar eq of lipid to the Nt17 peptide solution (60 μm final concentration) and incubating the peptide/LUV samples at room temperature for 1 h before CD measurements.

### Preparation and characterization of PFFs

To prepare Httex1 PFFs, a 300 μm solution of Httex1 was prepared by dissolving lyophilized proteins in 50 mm Tris and 150 mm NaCl, pH 7.5. The resulting solution was centrifuged at 14,000 × *g* (10 min at 4 °C), and the supernatant was then incubated at 37 °C under constant agitation at 850 rpm on an orbital shaker for 5 days. The generated fibrils were then briefly sonicated (40% amplitude, one pulse for 5 s) and then aliquoted and stored at −20 °C. Amyloid formation (before and after sonication) was assessed by TEM analysis.

### Primary neuron culture preparation and treatment with preformed fibrils

Primary striatal neuronal cultures were prepared from P0 Sprague–Dawley rats as described previously (38). Briefly, dissected tissues were dissociated with papain and triturated using a glass pipette. After centrifugation at 400 × *g* for 2 min, the cells were plated in minimum essential medium, 10% horse serum onto poly-l-lysine (Sigma)-coated coverslips (glass 12 mm, VWR) at 1.5 × 100,000 cells/ml. The medium was changed after 4 h to Neurobasal/B27 medium. After 7 days *in vitro*, the neurons were treated with Httex1 PFFs. The concentrations and durations of treatment are indicated in the respective figure legends.

### Western blot analysis of purified Httex1 protein

Protein samples were separated at 180 V on 15% polyacrylamide 1.5-mm-thick gels for 1 h. The proteins were transferred onto nitrocellulose membranes (Omnilab SA) using a semidry transfer system (Bio-Rad) under constant current (500 mA), and the membranes were blocked for 1 h at room temperature with Odyssey blocking buffer (Li-COR Biosciences) diluted 3-fold in PBS buffer. The membranes were probed with the relevant primary antibodies for 4 h at room temperature. (MAB5492, 1:2000 (Millipore); MW8, 1:1000 (Developmental Studies Hybridoma Bank)). After three washes with PBS buffer containing 0.1% (v/v) Tween 20 (Fluka), the membrane was incubated with secondary goat anti-mouse Alexa 680 and goat anti-rabbit Alexa 800 (dilution 1:2000; Invitrogen). The membranes were then washed three times with PBS buffer containing 0.01% (v/v) Tween 20 and once with PBS buffer, and then they were scanned on a LI-COR scanner at 700 and 800 nm.

### Immunocytochemistry and imaging of primary neurons

As described previously (38), primary neurons were washed with PBS, fixed with 4% paraformaldehyde in PBS, and then incubated in blocking buffer (3% BSA, 0.02% saponin in PBS) for 1 h at room temperature. Coverslips were then incubated overnight at 4 °C in blocking buffer comprising primary antibodies (MW8, Developmental Studies Hybridoma Bank; MAP2, Cell Signaling). Subsequently, the coverslips were washed five times with PBS and incubated in blocking buffer containing the appropriate Alexa Fluor–conjugated secondary antibodies (Invitrogen) as well as the nuclear stain Hoechst 33342 for 1 h at room temperature. After five washes with PBS, the coverslips were washed once with double-distilled water and then mounted on glass slides with DABCO mounting medium (Sigma-Aldrich). The neurons were then imaged using an LSM700 confocal microscope (Carl Zeiss). Image analysis was performed using Zen 2012 SP1 (black edition, Carl Zeiss) and FIJI software.

## Author contributions

S. M. D., F. S. R., M.-B. F., A. C., U. C., G. D., and H. A. L. conceptualization; S. M. D., F. S. R., M.-B. F., A. C., and U. C. data curation; S. M. D., F. S. R., M.-B. F., A. C., and U. C. formal analysis; S. M. D., M.-B. F., and H. A. L. methodology; S. M. D., F. S. R., M.-B. F., A. C., U. C., and G. D. writing-original draft; H. A. L. resources; H. A. L. supervision; H. A. L. funding acquisition; H. A. L. investigation; H. A. L. project administration; H. A. L. writing-review and editing.

## Supplementary Material

Supporting Information

## Footnotes

3 The abbreviations used are:

HD: Huntington′s disease
TCEP: tris(2-carboxyethyl)phosphine
PTFE: polytetrafluoroethylene
POPG: 1-palmitoyl-2-oleoyl-*sn*-glycero-3-phospho-(1′-racglycerol)
LUV: large unilamellar vesicle
PFF: preformed fibrillar species
PTM: post-translational modification
NBz: *N*-acyl-benzimidazolinone(s)
MPAA: 4-mercaptophenylacetic acid
NCL: native chemical ligation
ESI: electrospray ionization
TEM: transmission EM
AFM: atomic force microscopy
GdnHCl: guanidine hydrochloride
MeONH_4_Cl: methoxyaminehydrochloride
VA-044: 2–2′-azobis[2-(2-imidazolin-2-yl)propane] dihydrochloride
PMSF: phenylmethanesulfonyl fluoride
APTES: (3-aminopropyl)triethoxysilane.
