# Supplementary material for: N-terminal Huntingtin (Htt) phosphorylation is a molecular switch regulating Htt aggregation, helical conformation, internalization, and nuclear targeting
Source: J Biol Chem. 2018 Sep 5;293(48):18540–58. doi: 10.1074/jbc.RA118.004621 (PMC6290154; doi:10.1074/jbc.RA118.004621)
Supplement: Supporting Information [file supp_293_48_18540__index.html]

N-terminal Huntingtin (Htt) phosphorylation is a molecular switch regulating Htt aggregation, helical conformation, internalization, and nuclear targeting — PTMs as switches of Huntingtin structure and aggregation — N-terminal Huntingtin (Htt) phosphorylation is a molecular switch regulating Htt aggregation, helical conformation, internalization, and nuclear targeting — PTMs as switches of Huntingtin structure and aggregation — Supporting Information 

# N-terminal Huntingtin (Htt) phosphorylation is a molecular switch regulating Htt aggregation, helical conformation, internalization, and nuclear targeting

## Supporting Information

- Supplemental Figures - Supplemental Figures
