# Supplementary material for: N-terminal Huntingtin (Htt) phosphorylation is a molecular switch regulating Htt aggregation, helical conformation, internalization, and nuclear targeting
Source: J Biol Chem. 2018 Sep 5;293(48):18540–58. doi: 10.1074/jbc.RA118.004621 (PMC6290154; doi:10.1074/jbc.RA118.004621)
Supplement: Supporting Information [file supp_RA118.004621_139061_1_supp_186009_pdk39x.pdf]

N-terminal Huntingtin (Htt) phosphorylation is a molecular switch regulating Htt aggregation, helical conformation, internalization, and nuclear targeting

Sean M. DeGuire,[a] Francesco. S. Ruggeri,[b†] Mohamed-Bilal Fares,[a†] Anass Chiki, [a] Urszula Cendrowska,[b] Giovanni Dietler,[b] and Hilal A. Lashuel[a*]

[a.] Laboratory of Molecular and Chemical Biology of Neurodegeneration, Brain Mind Institute, École Polytechnique Fédérale de Lausanne (EPFL), CH-1015 Lausanne, Switzerland.

[b.] The Laboratory of the Physics of Living Matter, Institute of Physics of Biological Systems, École Polytechnique Fédérale de Lausanne (EPFL), CH-1015 Lausanne, Switzerland.

Running title: PTMs as switches of Huntingtin structure and aggregation

† These authors contributed equally to this work

* Corresponding author: hilal.lashuel@epfl.ch

Keywords: Huntington's disease, aggregation, phosphorylation, post-translational modification, neurodegenerative disease, Htt protein, amyloid, phosphomimetic, Httex1

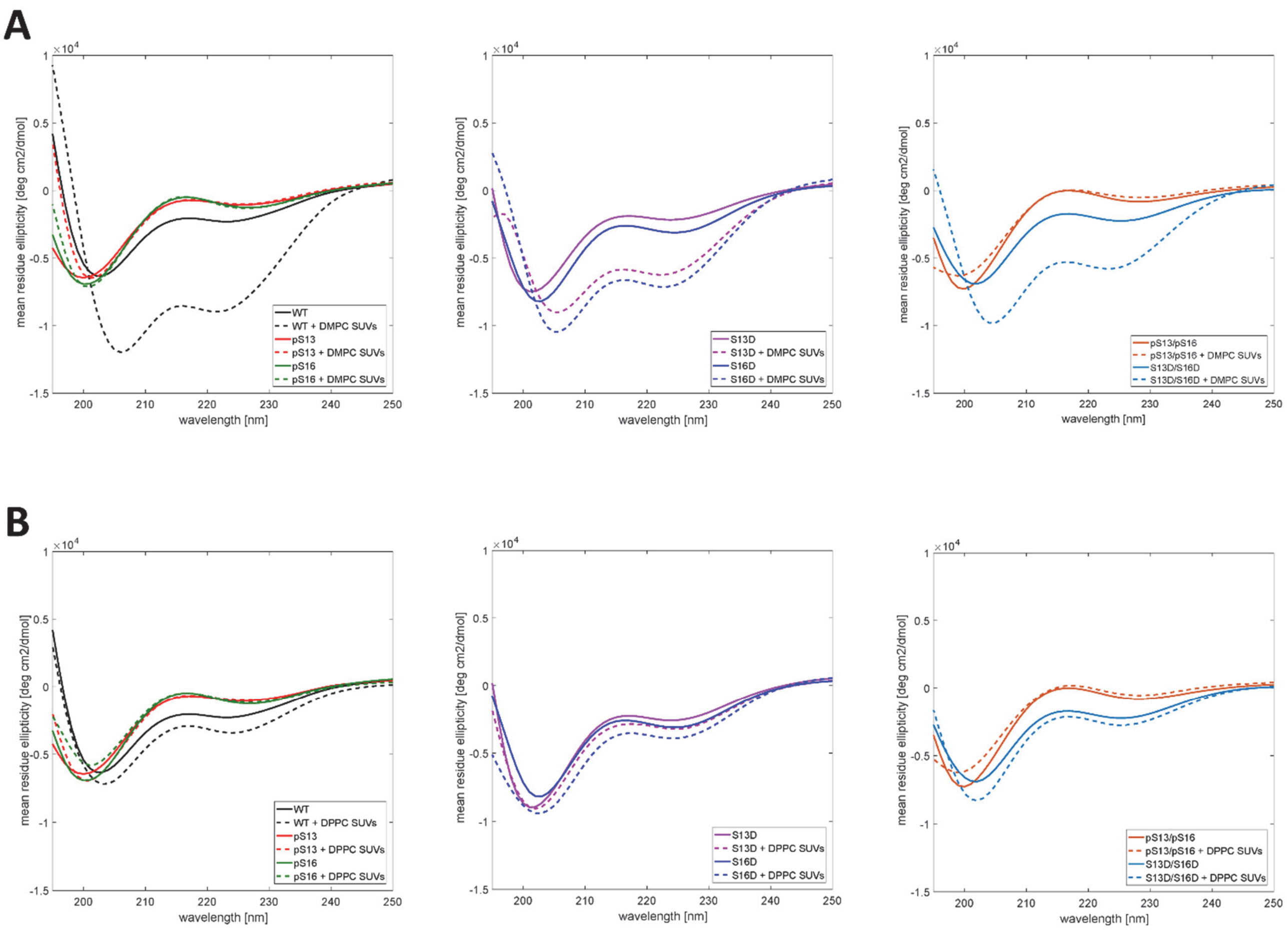

Supplemental Figure 1. Far-UV CD spectra of WT, pS13, pS16, pS13/pS16, S13D, S16D and S13D/S16D Nt17 in the presence and absence of dimyristoylphosphatidylcholine (DMPC) **(A)** or dipalmitoylphosphatidylcholine (DPPC) **(B)**.

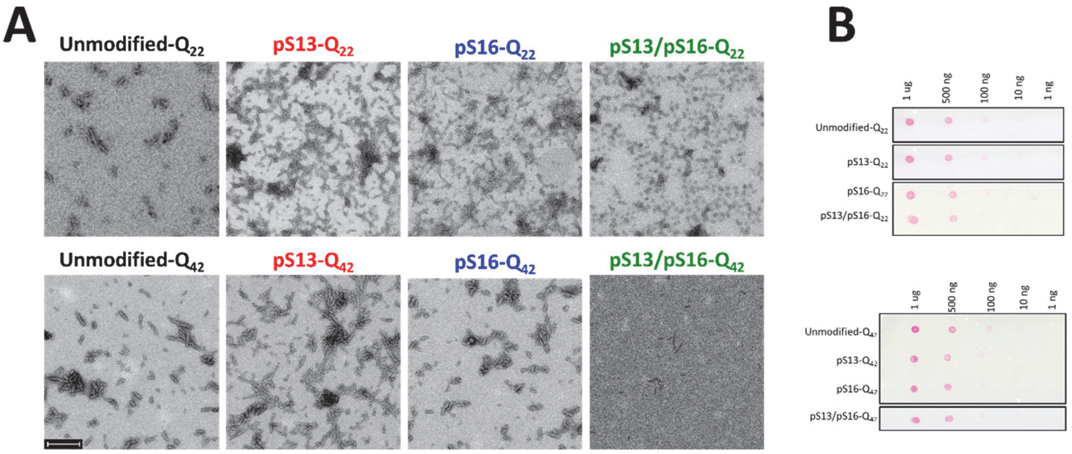

22Q	[µM]
Unmodified	100
pS13	94
pS16	78
pS13/pS16	69

42Q	~[µM]
Unmodified	100
pS13	72
pS16	62
pS13/pS16	70

Supplemental Figure 2. (A) TEM analysis of unmodified or phosphorylated Httex1 PFFs after sonication (Scale bar = 200 nm) and **(B)** Dot blot analysis with Ponceau staining for the relative quantification of PFFs prior to cellular treatment and concentration determination (table in right panel).

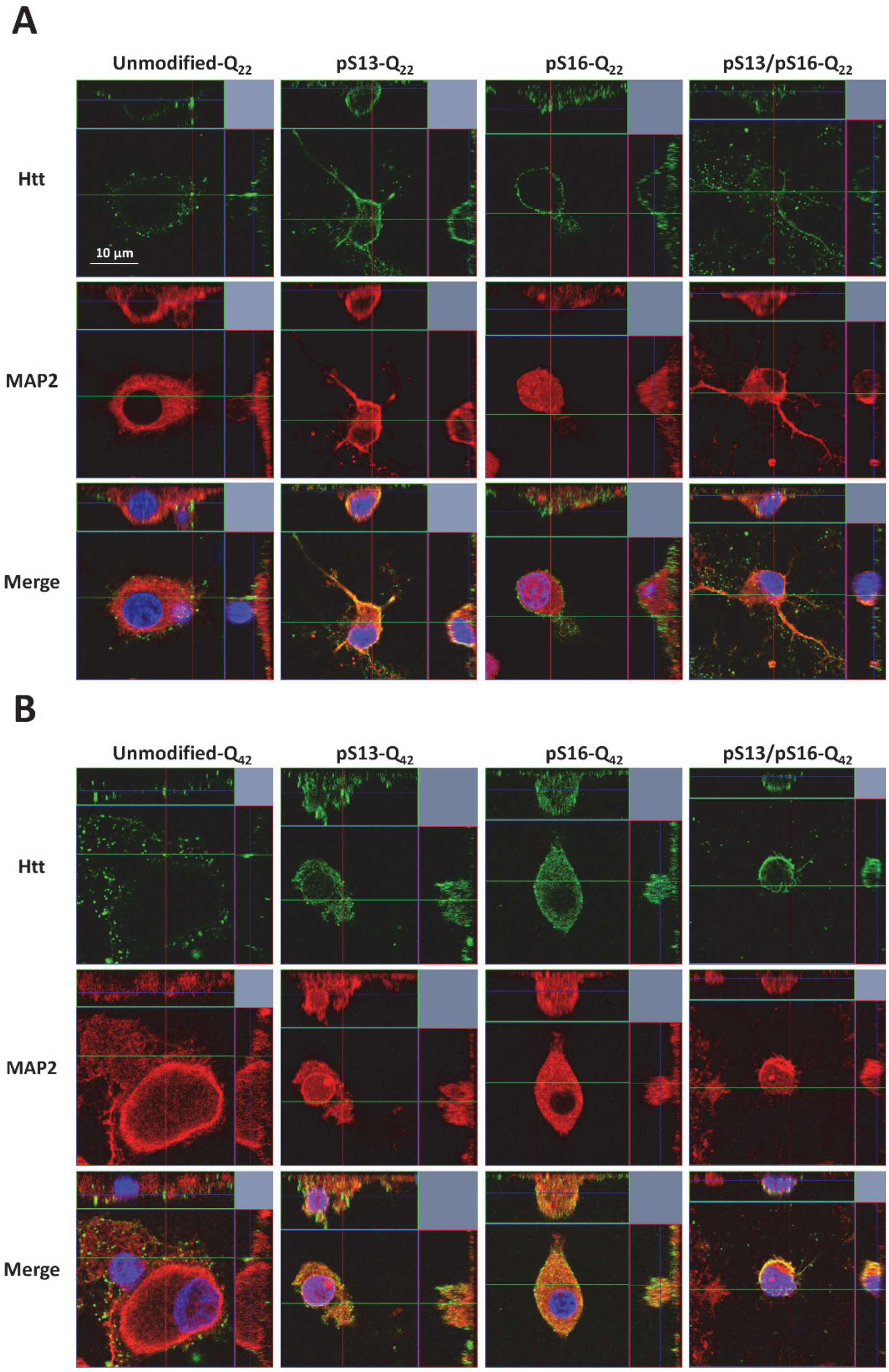

Supplemental Figure 3: Orthogonal projections of individual channels of neurons shown in Figures 10B and 11B are presented, to allow better visualization of internalized aggregates of PTM Q22 (A) or Q42 (B) PFFs. In (A) and (B), the uppermost row corresponds to immuno-staining using the MW8 anti-total Htt antibody, the middle row corresponds to MAP2 staining to reveal neuronal cell bodies, and the lowest row shows merged images of both channels, in addition to Hoechst-33342staining to reveal neuronal nuclei. Note that images showing the merged channels in (A) and (B) are reused from figure 10B and figure 11B, respectively, as these correspond to the same neurons presented in those figures.
